# Analysis of risk factors for postoperative mortality in acute type A aortic dissection patients under different critical levels

**DOI:** 10.1038/s41598-023-35351-w

**Published:** 2023-05-19

**Authors:** Xiyu Zhu, Junxia Wang, Hoshun Chong, Yi Jiang, Fudong Fan, Jun Pan, Hailong Cao, Yunxing Xue, Dongjin Wang, Qing Zhou

**Affiliations:** 1 Department of Cardio-Thoracic Surgery, Nanjing Drum Tower Hospital, The Affiliated Hospital of Nanjing University Medical School, No. 321 Zhongshan Rd, Nanjing, 210008 Jiangsu China; 2 Institute of Cardiothoracic Vascular Disease, Nanjing University, Nanjing, China; 3 Department of Cardio-Thoracic Surgery, Nanjing Drum Tower Hospital Clinical College of Nanjing Medical University, Nanjing, China

**Keywords:** Outcomes research, Cardiovascular biology

## Abstract

We built up a risk stratification model to divide acute type A aortic dissection (aTAAD) patients into low- and high-risk groups, further, to evaluate the risk factors for postoperative mortality. A total of 1364 patients from 2010 to 2020 in our center were retrospectively analyzed. More than twenty clinical variables were related with postoperative mortality. The postoperative mortality of the high-risk patients was doubled than the low-risk ones (21.8% vs 10.1%). The increased operation time, combined coronary artery bypass graft, cerebral complications, re-intubation, continuous renal replacement therapy and surgical infection were risk factors of postoperative mortality in low-risk patients. In addition, postoperative lower limbs or visceral malperfusion were risk factors, axillary artery cannulation and moderate hypothermia were protective factors in high-risk patients. A scoring system for quick decision-making is needed to select appropriate surgical strategy in aTAAD patients. For low-risk patients, different surgical treatments can be performed with similar clinical prognosis. Limited arch treatment and appropriate cannulation approach are crucial in high-risk aTAAD patients.

## Introduction

Aortic dissection, especially acute type A aortic dissection (aTAAD), is the most lethal cardiovascular disease. The mortality of aTAAD patients has decreased in the high-volume centers which can be attributed to improved surgical techniques and intra-operative organ protective procedures^1^. A series of studies have reported that advanced age, preoperative severe conditions, malperfusion syndrome, massive blood transfusion and postoperative renal failure were independent risk factors of postoperative mortality in aTAAD patients^2–6^. The preoperative condition and the surgical treatment are two key elements which influenced the postoperative complications and mortality in aTAAD patients. Thus, it is necessary to build up a scoring system to make the critical classification as soon as possible after admission.

A total of seven mortality related predictive models for aTAAD patients have been proposed in the past two decades^7–13^. The area under the receiver operating characteristic curve (AUROC) was range from 0.66 to 0.86. Some limitations of the existing models should be mentioned: (1) the scores are developed based on the small sample size; (2) the variables are heterogeneous; (3) organ perfusion status is not taken into consideration. On the other hand, different surgical techniques have been reported alternative and effective to patients under stable condition. Whether these techniques are also suitable for critically ill patients still remain uncertain.

In this study, we constructed a new scoring system for aTAAD patients and analyze the risk factors of mortality in different preoperative status patients in order to find out the optimal surgical procedure.

## Results

### Risk factors of postoperative mortality in aTAAD patients

**Figure 1:**
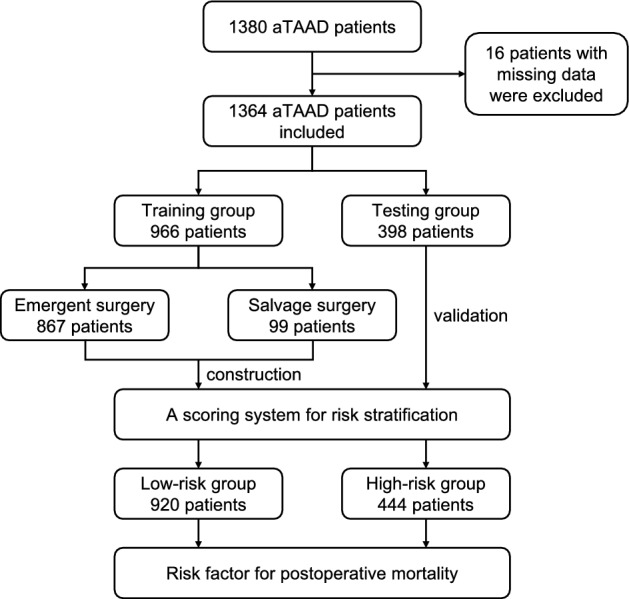
Flowchart diagram of patients. Total of 1380 aTAAD patients were enrolled, sixteen patients were excluded because of incomplete data. The data of the remaining 1364 patients were used for further analysis.

There were 1380 aTAAD patients admitted in our center from Jan 2010 to Dec 2020, sixteen of them were excluded due to data missing. Clinical characteristics of the remaining 1364 patients including preoperative, intraoperative and postoperative variables were collected and listed in Supplemental Table S1. The flow chart of this study was shown in Fig. 1.

**Figure 2:**
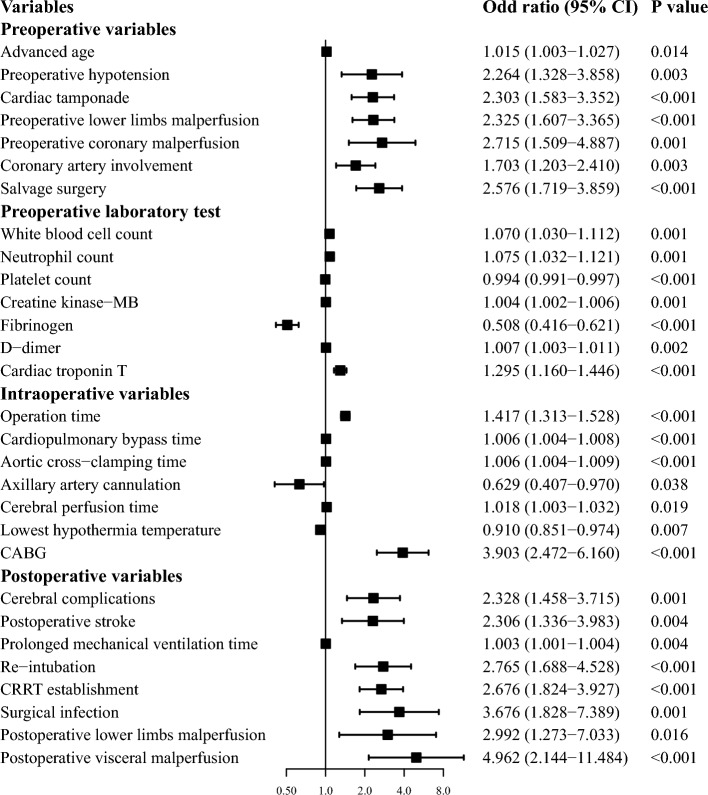
Risk factors for postoperative mortality in aTAAD patients. Clinical variables were divided into four parts as preoperative variables, preoperative laboratory test, intraoperative variables and postoperative variables. Odds ratio and 95% CI were represented by forest plot.

The overall postoperative mortality rate was 13.9%, after the preliminary analysis, we found it was related with more than twenty perioperative clinical variables listed as follows, (1) preoperative variables: advanced age (OR = 1.015, P = 0.014), hypotension (OR = 2.264, P = 0.003), cardiac tamponade (OR = 2.303, P < 0.001), preoperative lower limbs malperfusion (OR = 2.325, P < 0.001), preoperative coronary malperfusion (OR = 2.715, P = 0.001), coronary artery involvement (OR = 1.703, P = 0.003), salvage surgery (OR = 2.576, P < 0.001); (2) intraoperative variables: operation time (OR = 1.417, P < 0.001), CPB time (OR = 1.006, P < 0.001), aortic cross-clamping (ACC) time (OR = 1.006, P < 0.001), axillary artery cannulation (OR = 0.629, P = 0.038), cerebral perfusion time (OR = 1.018, P < 0.001), lowest hypothermia temperature (OR = 1.099, P = 0.007), CABG (OR = 3.903, P = 0.007); (3) postoperative variables: cerebral complications (OR = 2.328, P = 0.001), postoperative stroke (OR = 2.306, P = 0.004), prolonged mechanical ventilation time (OR = 1.003, P = 0.004), re-intubation (OR = 2.765, P < 0.001), CRRT establishment (OR = 2.676, P < 0.001), surgical infection (OR = 3.676, P = 0.001), postoperative lower limbs malperfusion (OR = 2.992, P = 0.016) and postoperative visceral malperfusion (OR = 4.962, P < 0.001); (4) laboratory test: white blood cell count (OR = 1.070, P = 0.001), neutrophil count (OR = 1.075, P = 0.001), platelet count (OR = 0.994, P < 0.001), creatine kinase-MB (OR = 1.004, P = 0.001), cardiac troponin T (OR = 1.295, P < 0.001), fibrinogen (OR = 0.508, P < 0.001) and d-dimer (OR = 1.007, P < 0.001) (Fig. 2 and Supplemental Table S2).

### Construction of the critical stratification of the aTAAD patients

**Figure 3:**
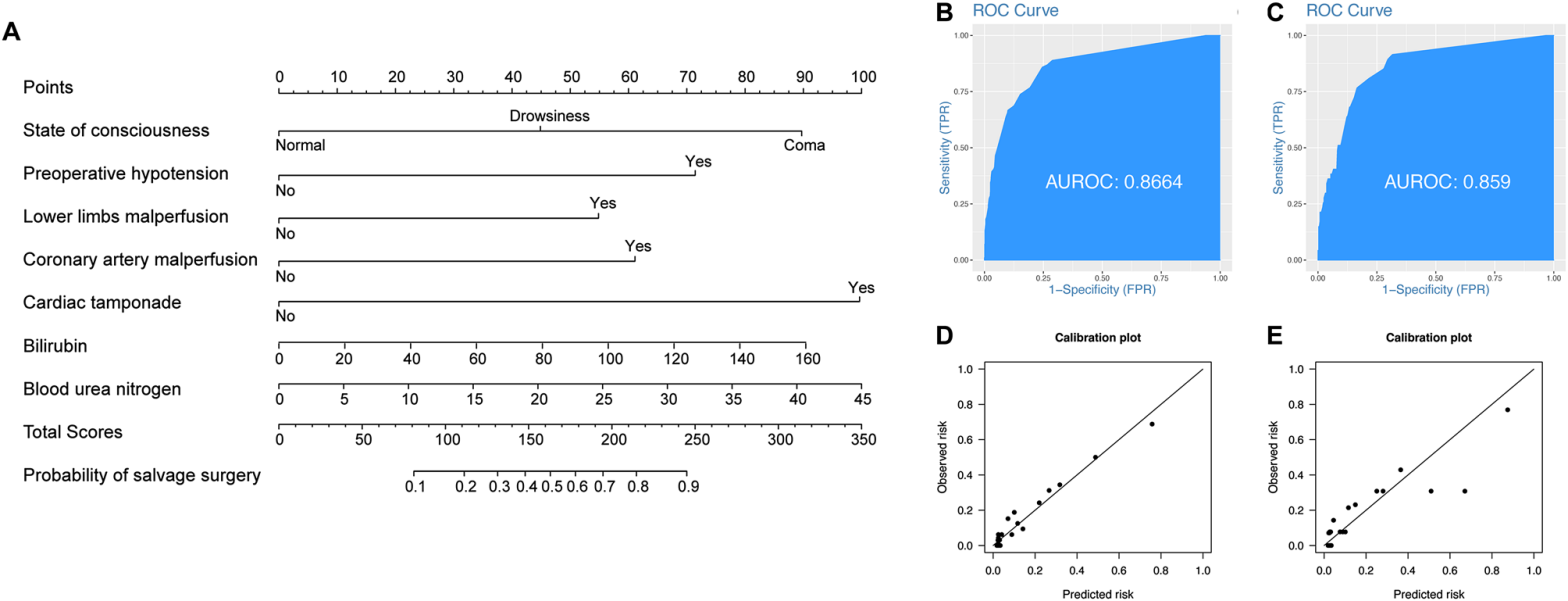
Critical stratification model of the aTAAD patients. (**A**) Nomogram of the critical stratification model of the aTAAD patients. (**B**,**C**) ROC curve of Model 2 a in training and testing group. (**D**,**E**) Calibration plot of Model 2 in training and testing group.

All patients were divided into training group (966 patients, 70.8% of total patients) and testing group (398 patients, 29.2% of total patients). No difference was found in preoperative variables between two groups except the ratio of hypertension, Marfan syndrome and hypotension (Supplemental Table S3). The variable “salvage surgery” was selected according to the results of the Spearman’s correlation (Supplemental Fig. S1) to redistribute patients into “Emergent surgery” and “Salvage surgery” group (Supplemental Table S4). Independent risk factors were selected by logistics regression analysis (Supplemental Table S5) and the best subset was evaluated by the value of Mallows’s Cp and adjusted R^2^ (Supplemental Fig. S2). The model 2 was chosen because of the lower Cp value and larger AUROC (0.87 vs 0.85) and was visualized by nomogram (Fig. 3A) with alternative calibration and discrimination separately (Fig. 3B–E). The Youden’s index of the model was 70 points, the sensitivity and the specificity were 87% and 74%.

### Risk factors of postoperative mortality in low- and high-risk patients

There were 920 low-risk and 444 high-risk patients according to the critical stratification model in our study. The postoperative mortality of the low- and high-risk patients were 10.1% and 21.8% separately. Difference between survival and deceased patients in low- and high-risk patients were listed in the Supplemental Table S6. For low-risk patients, increased operation time, CPB time, ACC time and combined CABG procedure were found with poor prognosis. Patients died after surgery also experienced more cerebral complications (6.2% vs 11.8%, P = 0.048), increased mechanical ventilation time (22.0 vs 41.5 h, P = 0.001), re-intubation rate (5.4% vs 15.1%, P = 0.001), CRRT rate (8.6% vs 25.8%, P < 0.001) and more surgical infection (2.1% vs 7.5%, P = 0.007). For high-risk patients, increased operation time, CPB time, ACC time, combined CABG procedure and lower hypothermia temperature were found with poor prognosis. These patients experienced more cerebral complications (7.8% vs 16.5%, P = 0.013), increased mechanical ventilation time (31.0 vs 66.0 h, P = 0.001), re-intubation rate (4.6% vs 11.3%, P = 0.019), more surgical infection (1.7% vs 6.2%, P = 0.028), postoperative lower limbs (2.3% vs 8.2%, P = 0.011) and visceral (1.7% vs 8.2%, P = 0.004) malperfusion. We also found that axillary artery cannulation (P = 0.048) and MiTAR procedure (P < 0.001) would improve the prognosis in high-risk patients.

Difference of the intraoperative variables showed that decreased cerebral perfusion (86.1% vs 79.7%, P = 0.003), especially anterograde cerebral perfusion, was found in high-risk patients. The rate of TAR (36.4% vs 28.6%, P = 0.005) and stent implantation (83.3% vs 76.4%, P = 0.009) were also decreased in high-risk patients.

Univariate analysis showed that, in terms of low-risk patients, the operation time (1.512 [1.353–1.689]), CPB time (1.005 [1.003–1.008]), ACC time (1.007 [1.003–1.011]), combined CABG procedure (3.116 [2.026–4.792]), cerebral complications (2.041 [1.024–4.070]), re-intubation (3.080 [1.619–5.857]), CRRT establishment (3.704 [2.192–6.257]) and surgical infection (3.878 [1.564–9.614]) were risk factors for postoperative mortality (Supplemental Table S7). On the other hand, lower hypothermia temperature (1.122 [1.021–1.233]), postoperative lower limbs malperfusion (3.809 [1.391–10.431]) and postoperative visceral malperfusion (5.109 [1.728–15.102]) were risk factors, axillary artery cannulation (0.464 [0.236–0.914]) was protective factors for high-risk patients (Supplemental Table S7).

## Patients and methods

### Patients and preoperative data collection

This study was approved by the Medical Ethics Committee of Nanjing Drum Tower Hospital (approval number: 2022-157) and followed the Declaration of Helsinki. Written informed consents were obtained from all participants.

ATAAD patients admitted from Jan 2010 to Dec 2020 in our center were included. Patients were diagnosed with contrast-enhanced CT scan in local hospital or the emergency unit of our hospital after dissection onset. The classification of aortic dissection was based on the Debakey classification. Preoperative data were collected as the following: demographic data (age, gender and body mass index); concomitant diseases (such as history of hypertension, diabetes, coronary artery disease, chronic obstructive pulmonary disease, atrial fibrillation, stroke and end stage renal disease); lifestyles (history of smoke and alcohol abuse); genetic components (family history of aortic aneurysm, dissection or Marfan’s syndrome); surgical history of coronary artery bypass graft (CABG), aortic valve replacement or thoracic endovascular aortic/aneurysm repair; clinical symptoms (including preoperative hypotension, cardiac tamponade, malperfusion syndrome and conscious status). The malperfusion syndrome was defined as malperfusion secondary to the dissection, including cerebral, limbs, visceral, coronary and renal malperfusion. The entry site of the intimal tear was evaluated by CT scan. Preoperative coronary artery involvement was evaluated by clinical symptom, preoperative electrocardiograph and echocardiography and confined by intraoperative exploration. Preoperative laboratory tests results were collected in different aspects.

### Surgical treatments

All patients in this study received surgical treatment. Cardiopulmonary bypass (CPB) was established according to the condition of the peripheral arteries and the cerebral protection method. The root treatment was decided by the modified root classification of aortic dissection^14^. Arch repair strategy was made according to the intimal tear, arch dissection, intraoperative exploration and surgeon selection. Four strategies of arch repair, such as hemi-arch replacement, total arch replacement (TAR), modified “in situ” TAR (MiTAR), and fenestrated stent implantation, were commonly used and were described in detail in our former studies^15–17^. Frozen elephant trunk (FET) was combined with techniques mentioned above to help aortic remodeling. Deep hypothermic circulatory arrest (DHCA) and cerebral protection were needed during arch repair in most of patients. CABG procedure was performed in patients with myocardial infraction or coronary artery dissection. Mitral valve repair, mitral valve replacement or tricuspid valve repair was performed if necessary.

### Postoperative managements

Postoperative death was recorded as the major clinical endpoint. Cerebral complications including stroke, hemiplegia and hemorrhage were proved by CT scan and neurological symptoms. Tracheotomy was performed in patients with mechanical ventilation over 1 week. Continuous renal replacement therapy (CRRT) was performed in patients with severe acute kidney injury after surgery. Second thoracotomy was performed if necessary, such as postoperative hemorrhage, cardiac tamponade and serious mediastina infection. Postoperative malperfusion syndrome was also recorded and treated accordingly.

### Statistical analysis

Numerical variables were described as mean ± standard deviation (SD) or median (quartile), categorical variables were described by count number with percentage. Statistical analysis was performed by using SPSS version 26.0. Continuous variables were compared by Student’s *t* test or Mann–Whitney *U* test according to the normality test. Categorical variables were compared with the Chi-square test, fisher’s exact was performed when sample size was less than five. The predictive model was conducted and visualized by the “Rms” package under the R language platform (version 4.0.3). In summary, patients were divided into two groups (training group and testing group) in the ratio of 7:3 through generation of the random numbers. The relationships between each preoperative variable were evaluated by the Spearman’s correlation analysis. Logistics regression analysis was performed to get the predictive variables according to the surgical status in training group. The optimal subset was selected by the “leaps” package and decided by the adjusted R^2^ and Cp value. The model was visualized by the “Rms” package. The total score of each patient was calculated by the “nomogramFormula” package. Calibration plot and receiver operating characteristic (ROC) curve were used to evaluate the calibration and discrimination of the model separately in the training and testing group. Youden’s index in both groups was calculated through the formula *J* = *sensitivity* + *specificity* − 1. P value less than 0.05 was considered significant difference.

## Comment

The postoperative mortality of aTAAD varies greatly among different centers (10–20%), a decline in mortality and morbidity were found in high volume center and experienced surgeon^18^. Several researchers have found that advanced age, preoperative severe conditions, malperfusion syndrome, massive blood transfusion and postoperative renal failure are independent risk factors for postoperative mortality in aTAAD patients^2–6^. However, the variation of preoperative condition and surgical decision brought uncertainty to the prognosis in aTAAD patients. A recent study has compared the predictive efficacy between GERAADA score (a scoring system based on the German Registry of Acute Aortic Dissection Type A) and EuroSCORE II (a risk evaluation system for cardiac operation) in aTAAD patients. The AUROC for postoperative mortality is 0.550 and 0.799 respectively^19^. Another small sample study reported the poor prediction capacity (AUROC = 0.566) of EuroSCORE II in aTAAD patients^20^. Thus, this study aims to construct a risk stratification model, based on the preoperative variables, to evaluate the clinical outcome of different surgical treatments on aTAAD patients.

In this study, we found that the postoperative mortality is associated with more than twenty clinical variables, some of which were consistent with previous studies, such as advanced age^12,21^, preoperative hypotension^21^, cardiac tamponade^21^, preoperative malperfusion syndrome of any organs^22–24^, elevated cardiac troponin T and D-dimer^25^. Apart from the preoperative variables, intraoperative and postoperative variables are also taken into consideration in our study. Salvage surgery, increased operation time, CPB time, AAC time, cerebral perfusion time, combined CABG, postoperative cerebral complications, prolonged mechanical ventilation time, re-intubation, CRRT establishment, surgical infection, postoperative lower limbs and visceral malperfusion were also risk factors for postoperative mortality among all aTAAD patients. Considering the influence of preoperative condition on the prognosis of patients, the results of the correlation test showed that the relationship between preoperative variables and mortality were highly correlated with the urgency of surgery, as “salvage surgery” and “emergent surgery” in this study (Fig. S1). Thus, we could evaluate the preoperative condition of patients through this model and divide them into low- and high-risk groups.

Researches have reported different clinical outcome of various root^26–29^ and arch treatments^15,30^ in aTAAD patients, however, the real-world results are still unclear because of the limited sample size and selection bias. In our center, we have reported the similar overall prognosis of different root treatment strategies^28,31^ and arch treatment strategies^15–17^. In this study, we found that the picture is quite different for patients with different preoperative condition. The low-risk patients share the similar clinical outcome under multiple surgical techniques dealing with the dissection lesion. In terms of critical preoperative status, the ratio of TAR decreases from 36.4 to 28.6% with an increase of mortality from 11.6 to 23.6%. Better surgical result was found in those high-risk patients underwent MiTAR surgery compared to other arch treatments, although there was no significant difference after removing the untreated and triple-branch stent implantation patients (P = 0.832). To our experience, it would be attributed to two main points: First, avoiding supra-arch vessels isolation and anastomosis could decrease operation time, CPB time, AAC time, DHCA time and intraoperative bleeding compared to TAR surgery^16^. Second, combined using of the FET would be helpful of early aortic remodeling^32^ compared to those without FET.

The preferred cannulation site for aTAAD surgery is also a focused clinical question in recent years^18^. Multiple retrospective researches have demonstrated that axillary artery cannulation is commonly performed in the Europe and the North America and can decrease the postoperative mortality in aTAAD patients^33,34^. In this study, for the low-risk patients, there is no difference of postoperative mortality in artery cannulation approaches of CPB (femoral artery, axillary artery, ascending aorta only or axillary-femoral artery). Axillary artery cannulation has shown protective effect in high-risk patients; however, the advantages of the axillary artery cannulation is not extended when axillary-femoral artery cannulation is used (12.8% vs 22.1%). Commonly, femoral artery cannulation is more efficient approach to establish CPB and has been widely used around the world^33^. Studies from STS database revealed that retrograde blood flow would increase the incidence of stroke^35^ and intraoperative organ malperfusion^36^. In this study, we found that high-risk patients with femoral artery cannulation experienced more postoperative stroke (5.9% vs 3.5%), hemiplegia (4.2% vs 2.3%), CRRT therapy (16.9% vs 9.3%) and lower limbs malperfusion (4.2% vs 1.2%) compared with axillary artery cannulation, despite no significant difference (Table S3).

In addition, postoperative mortality is highly related with increased operation time, CPB time, AAC time and concomitant CABG procedure (planned or unplanned) both in two groups in terms of intraoperative data which is similar to former studies^37^.

## Limitations

Several limitations of this study should be mentioned: (1) This is a retrospective and single center cohort study with limited sample size; (2) After incorporating more clinical variables, the predictive model will have higher sensitivity and specificity; (3) External validation from other centers is needed to examine the repeatability of this model, although internal validation have been performed; (4) Further researches are important to evaluate the influences of different surgical procedures and cannulation approaches on specific patients.

## Conclusions

The critical stratification model is useful to distinguish high-risk patients after admission immediately. For low-risk patients, different surgical treatments can be performed with similar clinical prognosis in high volume center. Limited and effective arch treatment and appropriate cannulation approach are crucial in high-risk aTAAD patients. Postoperative complications still need to be taken into consideration in all aTAAD patients.

## Supplementary Information

Supplementary Figure S1.

Supplementary Figure S2.

Supplementary Information.

## Data Availability

The datasets generated and analyzed during the current study are available from the corresponding author on reasonable request.
