# Supplementary figures and images for: Analysis of risk factors for postoperative mortality in acute type A aortic dissection patients under different critical levels
Source: Sci Rep. 2023 May 19;13:8107. doi: 10.1038/s41598-023-35351-w (PMC10199069; doi:10.1038/s41598-023-35351-w)

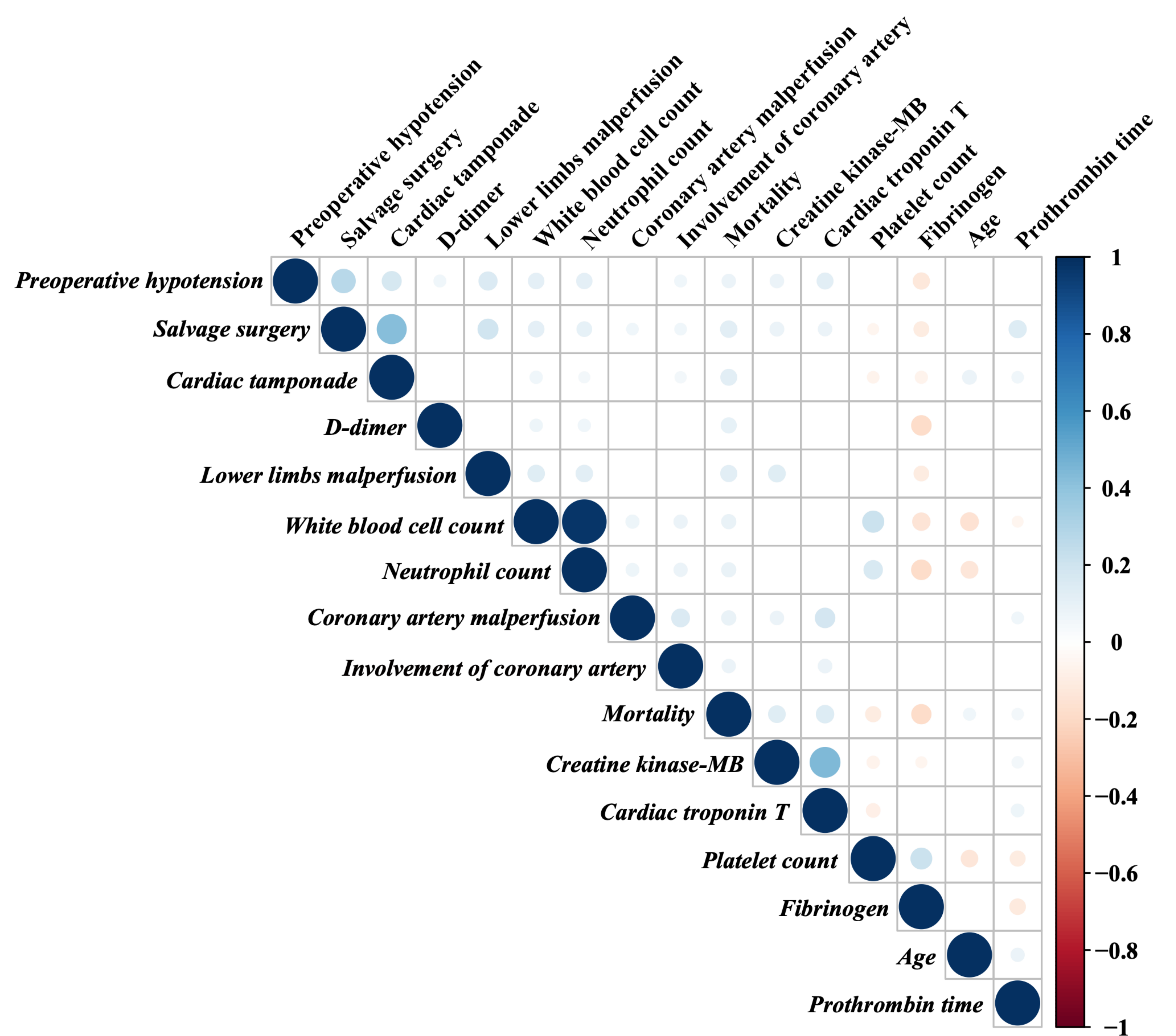

Supplement: Supplementary file 1 — Supplementary Figure S1. [file 41598_2023_35351_MOESM1_ESM.pdf]

A

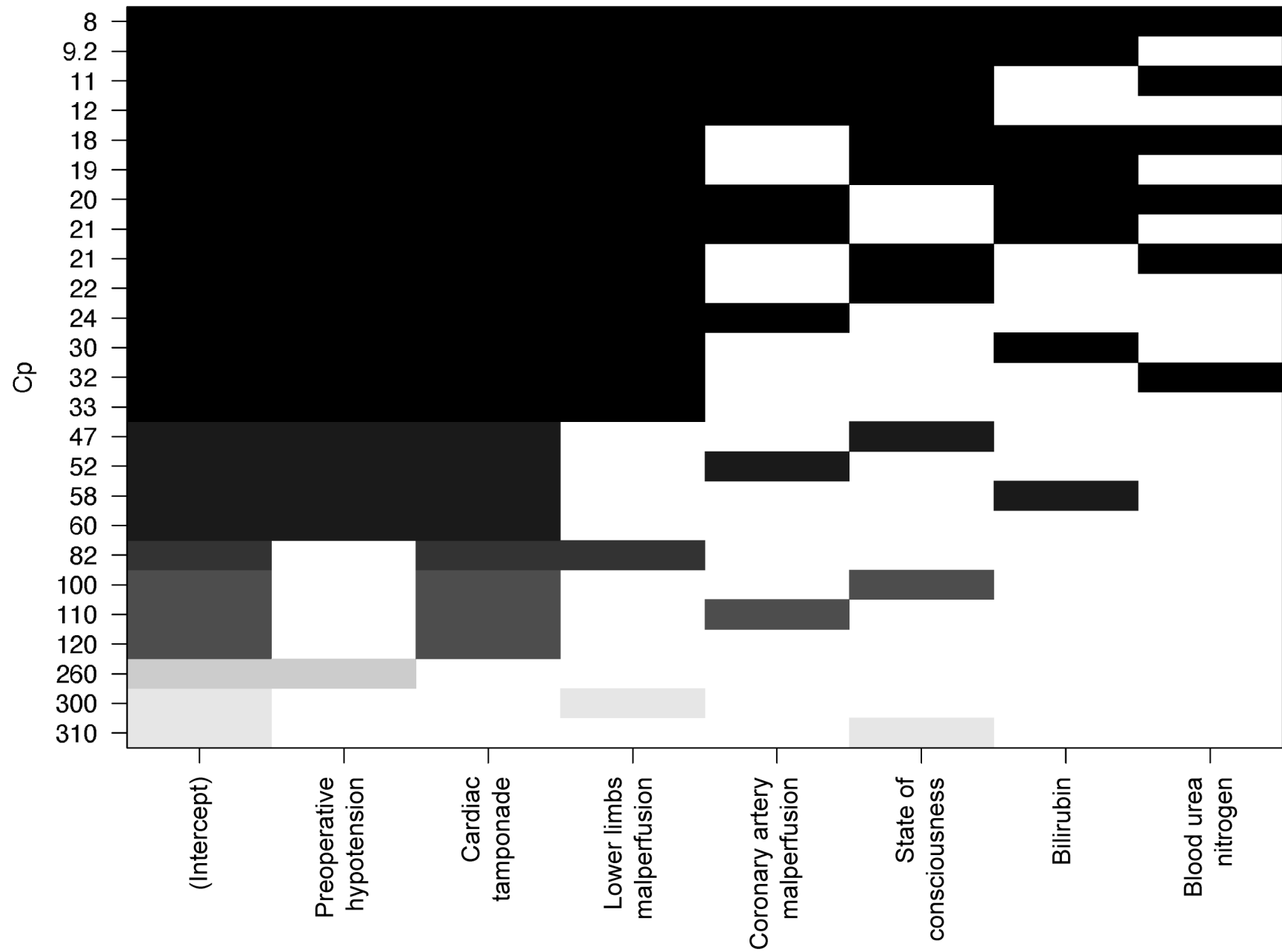

# B

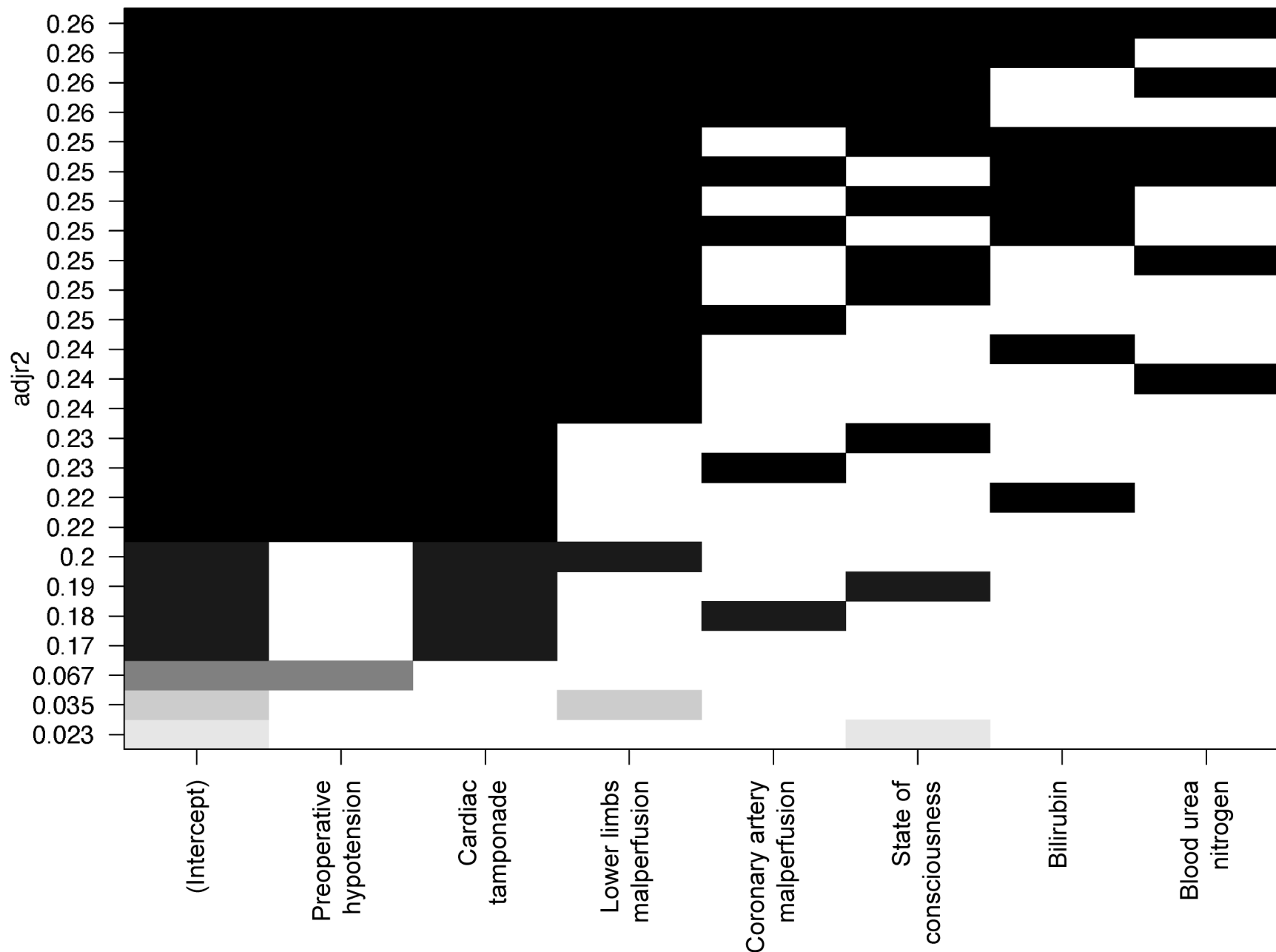

Supplement: Supplementary file 2 — Supplementary Figure S2. [file 41598_2023_35351_MOESM2_ESM.pdf]
