# Supplementary material for: Analysis of risk factors for postoperative mortality in acute type A aortic dissection patients under different critical levels
Source: Sci Rep. 2023 May 19;13:8107. doi: 10.1038/s41598-023-35351-w (PMC10199069; doi:10.1038/s41598-023-35351-w)
Supplement: Supplementary file 3 — Supplementary Information. [file 41598_2023_35351_MOESM3_ESM.docx]

**Supplemental materials**

**Analysis of risk factors for postoperative mortality in acute type A aortic dissection patients under different critical levels**

**Supplementary figure legends**

**Figure S1.** **Correlation of preoperative variables.** Spearman’s rank correlation coefficient was visualized by “corrplot” package. Blue circular means positive relevant and red circular means negative relevant. The correlation coefficient was listed in each intersection cell which relate to corresponding row and column items.

**Figure S2. Best subset selection of the risk stratification model.** A total of seven risk factors was selected to construct the best subset according to the logistics regression. The best subset was selected by the value of Mallows’s Cp (A) and adjusted R^2^ (B).

**Table S1. Clinical characters of aTAAD patients**

|  | **All patients**  **N=1364** | **Survival**  **N=1174** | **Death**  **N=190** | **P Value** |
| --- | --- | --- | --- | --- |
| **Preoperative variables** |  |  |  |  |
| Age (yrs old) | 53±13 | 53±13 | 55±13 | 0.02 |
| Gender (Male) | 1025 (75.1%) | 885 (75.4%) | 140 (73.7%) | 0.65 |
| Body mass index (kg/m^2^) | 25.7±4.0 | 25.7±4.0 | 25.7±4.0 | 0.98 |
| Hypertension | 977 (71.6%) | 839 (71.5%) | 138 (72.6%) | 0.80 |
| Marfan syndrome | 29 (2.1%) | 25 (2.1%) | 4 (2.1%) | >0.99 |
| Diabetes | 44 (3.2%) | 38 (3.2%) | 6 (3.2%) | >0.99 |
| CAD history | 30 (2.2%) | 24 (2.0%) | 6 (3.2%) | 0.29 |
| CABG history | 3 (0.2%) | 1 (0.1%) | 2 (1.1%) | 0.05 |
| TEVAR history | 31 (2.3%) | 26 (2.2%) | 5 (2.6%) | 0.61 |
| AVR history | 12 (0.9%) | 9 (0.8%) | 3 (1.6%) | 0.23 |
| Stroke history | 45 (3.3%) | 38 (3.2%) | 7 (3.7%) | 0.83 |
| Aortic aneurysm history | 19 (1.4%) | 17 (1.4%) | 2 (1.1%) | >0.99 |
| Family history of AD | 27 (2.0%) | 24 (2.0%) | 3 (1.6%) | >0.99 |
| COPD | 12 (0.9%) | 11 (0.9%) | 1 (0.5%) | >0.99 |
| Atrial fibrillation | 12 (0.9%) | 9 (0.8%) | 3 (1.6%) | 0.23 |
| End stage renal disease | 28 (2.1%) | 24 (2.0%) | 4 (2.1%) | >0.99 |
| Smoke abuse | 300 (22.0%) | 262 (22.3%) | 38 (20.0%) | 0.51 |
| Alcohol abuse | 214 (15.7%) | 186 (15.8%) | 28 (14.7%) | 0.75 |
| Cerebral ischemic attacks | 124 (9.1%) | 100 (8.5%) | 24 (12.6%) | 0.08 |
| Transient ischemic attacks | 100 (7.3%) | 85 (7.2%) | 15 (7.9%) | 0.76 |
| Stroke induced hemiplegia | 17 (1.2%) | 12 (1.0%) | 5 (2.6%) | 0.08 |
| Preoperative hypotension | 78 (5.7%) | 58 (4.9%) | 20 (10.5%) | 0.003 |
| Cardiac tamponade | 189 (13.9%) | 143 (12.2%) | 46 (24.2%) | <0.001 |
| Malperfusion syndrome |  |  |  |  |
| Visceral malperfusion | 54 (4.0%) | 42 (3.6%) | 12 (6.3%) | 0.11 |
| Coronary malperfusion | 62 (4.3%) | 44 (3.7%) | 18 (9.5%) | 0.001 |
| Lower limbs malperfusion | 197 (14.4%) | 149 (12.7%) | 48 (25.3%) | <0.001 |
| State of consciousness |  |  |  |  |
| Normal | 1340 (98.2%) | 1157 (98.6%) | 183 (96.3%) | 0.04 |
| Drowsiness | 17 (1.2%) | 11 (0.9%) | 6 (3.2%) |  |
| Coma | 7 (0.5%) | 6 (0.5%) | 1 (0.5%) |  |
| Intimal dissection entry site |  |  |  |  |
| Ascending aorta | 768 (56.3%) | 663 (56.5%) | 105 (55.3%) | 0.29 |
| Aortic arch | 368 (27.0%) | 309 (26.3%) | 59 (31.1%) |  |
| Descending aorta | 94 (6.9%) | 86 (7.3%) | 8 (4.2%) |  |
| Not found | 134 (9.8%) | 116 (9.9%） | 18 (9.5%) |  |
| Involvement of coronary artery | 276 (20.2%) | 222 (18.9%) | 54 (28.4%) | 0.003 |
| Salvage surgery | 146 (10.7%) | 107 (9.1%) | 39 (20.5%) | <0.001 |
| Laboratory tests |  |  |  |  |
| White blood cell count (10^9^/L) | 11.4 (9.1, 13.4) | 11.4 (9.0, 13.3) | 12.4 (10.8, 14.5) | <0.001 |
| Neutrophil count (10^9^/L) | 9.8 (7.6, 11.9) | 9.8 (7.6, 11.7) | 10.7 (9.3, 12.7) | <0.001 |
| Neutrophil ratio (%) | 85.8 (83.0, 90.0) | 85.8 (82.6, 90.0) | 85.8 (85.8, 89.2) | 0.06 |
| Red blood cell count (10^9^/L) | 3.93 (3.47, 4.44) | 3.93 (3.47, 4.47) | 3.83 (3.42, 4.35) | 0.04 |
| Hemoglobin (mg/dL) | 120 (105, 136) | 119 (105, 137) | 116 (101, 132) | 0.05 |
| Platelet count (10^9^/L) | 144 (108, 171) | 148 (111, 172) | 128 (88, 160) | <0.001 |
| Alanine transaminase (U/L) | 25.6 (17.8, 40.4) | 25.6 (17.1, 39.0) | 32.9 (23.1, 54.3) | <0.001 |
| Aspartate transaminase (U/L) | 38.0 (24.0, 62.0) | 36.0 (23.7, 54.2) | 61.9 (31.8, 87.0) | <0.001 |
| Lactate dehydrogenase (U/L) | 454.5 (348.0, 601.0) | 444.2 (339.0, 593.0) | 493.8 (429.0, 778.0) | <0.001 |
| Bilirubin (umol/L) | 16.2 (11.9, 22.0) | 16.2 (11.9, 22.1) | 16.9 (12.5, 22.0) | 0.13 |
| Creatinine (umol/L) | 81.2 (66.0, 108.2) | 81.2 (65.1, 106.9) | 97.2 (80.4, 137.9) | <0.001 |
| Blood urea nitrogen (mmol/L) | 7.3 (6.4, 8.2) | 7.30 (6.31, 8.14) | 7.68 (6.95, 8.88) | <0.001 |
| Creatine kinase-MB (U/L) | 10.5 (8.0, 18.0) | 10.5 (7.0, 16.0) | 18.0 (11.0, 23.3) | <0.001 |
| Cardiac troponin T (ug/L) | 0.025 (0.012, 0.094) | 0.025 (0.011, 0.082) | 0.070 (0.024, 0.182) | <0.001 |
| Brain natriuretic peptide (pg/mL) | 97.0 (45.0, 201.8) | 97.0 (44.1, 203.8) | 93.1 (54.5, 148.3) | 0.41 |
| Prothrombin time (s) | 13.1 (11.8, 14.2) | 12.8 (11.8, 14.0) | 15.3 (12.8, 15.5) | <0.001 |
| Fibrinogen (g/L) | 2.3 (1.7, 2.8) | 2.3 (1.8, 2.9) | 1.8 (1.4, 2.2) | <0.001 |
| D-dimer (mg/L) | 4.96 (3.98, 7.84) | 4.96 (3.82, 6.82) | 7.84 (5.96, 10.48) | <0.001 |
| **Intraoperative variables** |  |  |  |  |
| Operation time (hour) | 7.8±2.0 | 7.7±1.8 | 9.2±2.5 | <0.001 |
| CPB time (min) | 234.6±75.2 | 228.6±67.5 | 271.7±105.0 | <0.001 |
| ACC time (min) | 163.8±54.8 | 160.8±52.0 | 182.1±66.8 | <0.001 |
| Arterial cannulation approach |  |  |  |  |
| Ascending aorta | 20 (1.5%) | 16 (80%) | 4 (20%) | 0.16 |
| Femoral artery | 329 (24.1%) | 279 (84.8%) | 50 (15.2%) |  |
| Axillary artery | 276 (20.2%) | 248 (89.9%) | 28 (10.1%) |  |
| Axillary-Femoral artery | 739 (54.2%) | 631 (85.4%) | 108 (14.6%) |  |
| DHCA | 1345 (98.6%) | 1156 (98.5%) | 189 (99.5%) | 0.35 |
| Cerebral perfusion | 1146 (84.0%) | 991 (84.4%) | 155 (81.6%) |  |
| None | 218 (16.0%) | 183 (83.9%) | 35 (16.1%) | 0.51 |
| Anterograde | 1078 (79.0%) | 934 (86.6%) | 144 (13.4%） |  |
| Retrograde | 68 (5.0%) | 57 (83.8%) | 11 (16.2%) |  |
| Cerebral perfusion time (min) | 29.9±11.1 | 29.6±11.0 | 31.7±11.1 | 0.03 |
| Lowest hypothermia temperature (℃) | 21.1±2.4 | 21.1±2.4 | 20.6±2.4 | 0.004 |
| Root treatment |  |  |  |  |
| Untreated | 14 (1.0%) | 12 (85.7%) | 2 (14.3%) | 0.82 |
| Root repair | 1034 (75.8%) | 888 (85.9%) | 146 (14.1%) |  |
| Root replacement | 316 (23.2%) | 275 (87.0%) | 41 (12.9%) |  |
| Arch treatment |  |  |  |  |
| Untreated | 5 (0.4%) | 5（100%） | 0（0.0%） | 0.98 |
| Hemi-arch replacement | 273 (20%) | 234 (85.7%) | 39 (14.3%) |  |
| MiTAR | 160 (11.7%) | 139 (86.9%) | 21 (13.1%) |  |
| TAR | 462 (33.9%) | 393 (85.1%) | 69 (14.9%) |  |
| Triple-branch stent | 40 (2.9%) | 35 (87.5%) | 5 (12.5%) |  |
| Fenestrated stent | 424 (31.1%) | 368 (86.8%) | 56 (13.2%) |  |
| CABG | 93 (6.8%) | 60 (5.1%) | 33 (17.4%) | <0.001 |
| Planned CABG | 65 (69.9%) | 50 (80%) | 15 (20%) | <0.001 |
| Unplanned CABG | 28 (30.1%) | 10 (36.8%) | 18 (63.2%) |  |
| Mitral valve replacement | 7 (0.5%) | 5 (0.4%) | 2 (1.1%) | 0.25 |
| Mitral valve repair | 18 (1.3%) | 14 (1.2%) | 4 (2.1%) | 0.30 |
| Tricuspid valve repair | 22 (1.6%) | 19 (1.6%) | 3 (1.6%) | >0.99 |
| AFRA | 5 (0.4%) | 4 (0.3%) | 1 (0.5%) | 0.53 |
| **Postoperative variables** |  |  |  |  |
| Cerebral complications | 105 (7.7%) | 78 (6.6%) | 27 (14.2%) | 0.001 |
| Postoperative stroke | 76 (5.6%) | 57 (4.9%) | 19 (10.0%) | 0.007 |
| Postoperative hemiplegia | 28 (2.1%) | 23 (2.0%) | 5 (2.6%) | 0.58 |
| Mechanical ventilation time (hour) | 28.75 (13.5, 70.0) | 25.0 (25.0, 70.0) | 60.0 (60.0, 120.0) | <0.001 |
| Re-intubation | 86 (6.3%) | 61 (5.2%) | 25 (13.2%) | <0.001 |
| Tracheotomy | 56 (4.1%) | 47 (4.0%) | 9 (4.7%) | 0.69 |
| CRRT establishment | 167 (12.2%) | 122 (10.4%) | 45 (23.7%) | <0.001 |
| Secondary thoracotomy | 76 (5.6%) | 60 (5.1%) | 16 (8.4%) | 0.09 |
| Surgical infection | 36 (2.6%) | 23 (2.0%) | 13 (6.8%) | 0.001 |
| Lower limbs malperfusion | 25 (1.8%) | 17 (1.4%) | 8 (4.2%) | 0.02 |
| Visceral malperfusion | 23 (1.7%) | 13 (1.1%) | 10 (5.3%) | <0.001 |
| Gastrointestinal bleeding | 14 (1.0%) | 11 (0.9%) | 3 (1.6%) | 0.43 |

*Abbreviation:* AAC=aortic cross-clamping; AD=aortic dissection; AFRA=atrial fibrillation radiofrequency ablation; AVR=aortic valve replacement; CABG=coronary artery bypass graft; CAD=coronary artery disease; COPD=chronic obstructive pulmonary disease; CPB=cardiopulmonary bypass; CRRT= continuous renal replacement therapy; DHCA= deep hypothermic circulatory arrest; MiTAR= modified “*in situ*” total arch replacement; TAR= total arch replacement; TEVAR=thoracic endovascular aortic repair.

**Table S2. Univariate analysis of postoperative mortality in aTAAD patients**

| **Variable** | **Odds ratio** | **95% CI** | | **P value** |
| --- | --- | --- | --- | --- |
|  |  | **Lower** | **Upper** |  |
| Advanced age | 1.015 | 1.003 | 1.027 | 0.014 |
| Preoperative hypotension | 2.264 | 1.328 | 3.858 | 0.003 |
| Cardiac tamponade | 2.303 | 1.583 | 3.352 | <0.001 |
| Lower limbs malperfusion | 2.325 | 1.607 | 3.365 | <0.001 |
| Coronary malperfusion | 2.715 | 1.509 | 4.887 | 0.001 |
| Involvement of coronary artery | 1.703 | 1.203 | 2.410 | 0.003 |
| Salvage surgery | 2.576 | 1.719 | 3.859 | <0.001 |
| White blood cell count | 1.070 | 1.030 | 1.112 | 0.001 |
| Neutrophil count | 1.075 | 1.032 | 1.121 | 0.001 |
| Platelet count | 0.994 | 0.991 | 0.997 | <0.001 |
| Creatine kinase-MB | 1.004 | 1.002 | 1.006 | 0.001 |
| Fibrinogen | 0.508 | 0.416 | 0.621 | <0.001 |
| D-dimer | 1.007 | 1.003 | 1.011 | 0.002 |
| Cardiac troponin T | 1.295 | 1.160 | 1.446 | <0.001 |
| Operation time | 1.417 | 1.313 | 1.528 | <0.001 |
| CPB time | 1.006 | 1.004 | 1.008 | <0.001 |
| ACC time | 1.006 | 1.004 | 1.009 | <0.001 |
| Axillary artery cannulation | 0.629 | 0.407 | 0.970 | 0.038 |
| CABG | 3.903 | 2.472 | 6.160 | <0.001 |
| Cerebral perfusion time | 1.018 | 1.003 | 1.032 | 0.019 |
| Lowest hypothermia temperature | 1.099 | 1.027 | 1.175 | 0.007 |
| Cerebral complications | 2.328 | 1.458 | 3.715 | 0.001 |
| Postoperative stroke | 2.306 | 1.336 | 3.983 | 0.004 |
| Mechanical ventilation time | 1.003 | 1.001 | 1.004 | 0.004 |
| Re-intubation | 2.765 | 1.688 | 4.528 | <0.001 |
| CRRT establishment | 2.676 | 1.824 | 3.927 | <0.001 |
| Surgical infection | 3.676 | 1.828 | 7.389 | 0.001 |
| Postoperative lower limbs malperfusion | 2.992 | 1.273 | 7.033 | 0.016 |
| Postoperative visceral malperfusion | 4.962 | 2.144 | 11.484 | <0.001 |

*Abbreviation:* CPB=cardiopulmonary bypass; AAC=aortic cross-clamping; CABG=coronary artery bypass graft; CRRT= continuous renal replacement therapy.

**Table S3. Preoperative clinical characters of training and testing group patients**

|  | **All patients**  **N=1364** | **Training group**  **N=966** | **Testing group**  **N=398** | **P value** |
| --- | --- | --- | --- | --- |
| Age (yrs old) | 53±13 | 53±13 | 53±13 | 0.741 |
| Gender (Male) | 1025 (75.1%) | 731 (75.7%) | 294 (73.9%) | 0.491 |
| Body mass index (kg/m^2^) | 25.7±4.0 | 25.6±3.9 | 26.0±4.2 | 0.168 |
| Hypertension | 977 (71.6%) | 674 (69.8%) | 303 (76.1%) | 0.021 |
| Marfan syndrome | 29 (2.1%) | 26 (2.7%) | 3 (0.8%) | 0.036 |
| Diabetes | 44 (3.2%) | 35 (3.6%) | 9 (2.3%) | 0.239 |
| CAD history | 30 (2.2%) | 22 (2.3%) | 8 (2.0%) | 0.842 |
| CABG history | 3 (0.2%) | 3 (0.3%) | 0 (0.0%) | 0.560 |
| TEVAR history | 31 (2.3%) | 20 (2.1%) | 11 (2.8%) | 0.549 |
| AVR history | 12 (0.9%) | 7 (0.7%) | 5 (1.3%) | 0.348 |
| Stroke history | 45 (3.3%) | 31 (3.2%) | 14 (3.5%) | 0.868 |
| Aortic aneurysm history | 19 (1.4%) | 14 (1.4%) | 5 (1.3%) | 0.810 |
| Family history of AD | 27 (2.0%) | 18 (1.9%) | 9 (2.3%) | 0.670 |
| COPD | 12 (0.9%) | 11 (1.1%) | 1 (0.3%) | 0.198 |
| Atrial fibrillation | 12 (0.9%) | 10 (1.0%) | 2 (0.5%) | 0.376 |
| End stage renal disease | 28 (2.1%) | 18 (1.9%) | 10 (2.5%) | 0.528 |
| Smoke abuse | 300 (22.0%) | 216 (21.6%) | 84 (25.3%) | 0.616 |
| Alcohol abuse | 214 (15.7%) | 147 (15.2%) | 67 (16.8%) | 0.462 |
| Cerebral ischemic attacks | 124 (9.1%) | 85 (8.8%) | 39 (9.8%) | 0.604 |
| Transient ischemic attacks | 100 (7.3%) | 68 (7.0%) | 32 (8.0%) | 0.568 |
| Stroke induced hemiplegia | 17 (1.2%) | 14 (1.4%) | 3 (0.8%) | 0.422 |
| Preoperative hypotension | 78 (5.7%) | 46 (4.8%) | 32 (8.0%) | 0.021 |
| Cardiac tamponade | 189 (13.9%) | 130 (13.5%) | 59 (14.8%) | 0.546 |
| Malperfusion syndrome |  |  |  |  |
| Visceral malperfusion | 54 (4.0%) | 35 (3.6%) | 19 (4.8%) | 0.359 |
| Coronary malperfusion | 62 (4.3%) | 43 (4.5%) | 19 (4.8%) | 0.886 |
| Lower limbs malperfusion | 197 (14.4%) | 128 (13.3%) | 69 (17.3%) | 0.052 |
| State of consciousness |  |  |  |  |
| Normal | 1340 (98.2%) | 951 (98.4%) | 389 (97.7%) | 0.497 |
| Drowsiness | 17 (1.2%) | 10 (1.0%) | 7 (1.8%) |  |
| Coma | 7 (0.5%) | 5 (0.5%) | 2 (0.5%) |  |
| Intimal dissection entry site |  |  |  |  |
| Ascending aorta | 768 (56.3%) | 542 (56.1%) | 226 (56.8%) | 0.955 |
| Aortic arch | 368 (27.0%) | 262 (27.1%) | 106 (26.6%) |  |
| Descending aorta | 94 (6.9%) | 65 (6.7%) | 29 (7.3%) |  |
| Not found | 134 (9.8%) | 97 (10.0%) | 37 (9.3%) |  |
| Involvement of coronary artery | 276 (20.2%) | 201 (20.8%) | 75 (18.8%) | 0.416 |
| Salvage surgery | 146 (10.7%) | 99 (10.2%) | 47 (11.8%) | 0.441 |
| Laboratory tests |  |  |  |  |
| White blood cell count (10^9^/L) | 11.4 (9.1, 13.4) | 11.4 (9.1, 13.3) | 11.4 (8.9, 13.8) | 0.811 |
| Neutrophil count (10^9^/L) | 9.8 (7.6, 11.9) | 9.8 (7.6, 11.8) | 9.8 (7.3, 12.0) | 0.747 |
| Neutrophil ratio (%) | 85.8 (83.0, 90.0) | 85.8 (83.0, 89.7) | 85.9 (83.1, 89.9) | 0.939 |
| Red blood cell count (10^9^/L) | 3.93 (3.47, 4.44) | 3.93 (3.49, 4.43) | 3.93 (3.43, 4.45) | 0.765 |
| Hemoglobin (mg/dL) | 120 (105, 136) | 119.6 (105.0, 136.0) | 119.6 (103.0, 136.3) | 0.488 |
| Platelet count (10^9^/L) | 144.0 (108.0, 171.0) | 144.5 (109.8, 172.0) | 143.0 (105.0, 169.3) | 0.695 |
| Alanine transaminase (U/L) | 25.6 (17.8, 40.4) | 25.6 (17.1, 40.9) | 25.6 (19.0, 40.1) | 0.389 |
| Aspartate transaminase (U/L) | 38.0 (24.0, 62.0) | 39.0 (24.2, 61.9) | 35.3 (23.0, 60.3) | 0.648 |
| Lactate dehydrogenase (U/L) | 454.5 (348.0, 601.0) | 452.0 (345.5, 602.0) | 456.5 (351.8, 597.3) | 0.623 |
| Bilirubin (umol/L) | 16.2 (11.9, 22.0) | 16.2 (11.9, 22.1) | 16.2 (12.0, 21.5) | 0.872 |
| Creatinine (umol/L) | 81.2 (66.0, 108.2) | 81.2 (66.0, 105.0) | 83.0 (66.9, 121.1) | 0.091 |
| Blood urea nitrogen (mmol/L) | 7.3 (6.4, 8.2) | 7.3 (6.4, 8.2) | 7.3 (6.5, 8.2) | 0.165 |
| Creatine kinase-MB (U/L) | 10.5 (8.0, 18.0) | 10.5 (7.0, 18.0) | 10.5 (8.0, 18.0) | 0.334 |
| Cardiac troponin T (ug/L) | 0.025 (0.012, 0.094) | 0.025 (0.012, 0.090) | 0.025 (0.012, 0.129) | 0.564 |
| Brain natriuretic peptide (pg/mL) | 97.0 (45.0, 201.8) | 97.0 (44.0, 200.3) | 97.0 (47.9, 203.0) | 0.850 |
| Prothrombin time (s) | 13.1 (11.8, 14.2) | 13.0 (11.8, 14.1) | 13.2 (11.9, 14.3) | 0.721 |
| Fibrinogen (g/L) | 2.3 (1.7, 2.8) | 2.3 (1.7, 2.8) | 2.3 (1.7, 2.8) | 0.871 |
| D-dimer (mg/L) | 4.96 (3.98, 7.84) | 4.96 (3.95, 7.84) | 4.96 (3.99, 8.39) | 0.424 |
| Mortality | 190 (13.9%) | 133 (13.8%) | 57 (14.3%) | 0.797 |

*Abbreviation:* CAD=coronary artery disease; CABG=coronary artery bypass graft; TEVAR=thoracic endovascular aortic repair; AVR=aortic valve replacement; AD=aortic dissection; COPD=chronic obstructive pulmonary disease.

**Table S4. Preoperative clinical characters according to the surgical status in the training group patients**

|  | All patients  N=966 | Emergent surgery  N=867 | Salvage surgery  N=99 | P value |
| --- | --- | --- | --- | --- |
| Age (yrs old) | 53±13 | 53±13 | 54±14 | 0.38 |
| Gender (Male) | 731 (75.7%) | 654 (75.4%) | 77 (77.8%) | 0.63 |
| Body mass index (kg/m^2^) | 25.6±3.9 | 25.6±3.8 | 25.3±4.3 | 0.34 |
| Hypertension | 674 (69.8%) | 611 (70.5%) | 63 (63.6%) | 0.17 |
| Marfan syndrome | 26 (2.7%) | 22 (2.5%) | 4 (4.0%) | 0.33 |
| Diabetes | 35 (3.6%) | 32 (3.7%) | 3 (3.0%) | >0.99 |
| CAD history | 22 (2.3%) | 20 (2.3%) | 2 (2.0%) | >0.99 |
| CABG history | 3 (0.3%) | 1 (0.1%) | 2 (2.0%) | 0.03 |
| TEVAR history | 20 (2.1%) | 18 (2.1%) | 2 (2.0%) | >0.99 |
| AVR history | 7 (0.7%) | 7 (0.8%) | 0 (0.0%) | >0.99 |
| Stroke history | 31 (3.2%) | 27 (3.1%) | 4 (4.0%) | 0.55 |
| Aortic aneurysm history | 14 (1.4%) | 13 (1.5%) | 1 (1.0%) | >0.99 |
| Family history of AD | 18 (1.9%) | 16 (1.8%) | 2 (2.0%) | 0.71 |
| COPD | 11 (1.1%) | 11 (1.3%) | 0 (0.0%) | 0.62 |
| Atrial fibrillation | 10 (1.0%) | 8 (0.9%) | 2 (2.0%) | 0.27 |
| End stage renal disease | 18 (1.9%) | 15 (1.7%) | 3 (3.0%) | 0.42 |
| Smoke abuse | 216 (22.4%) | 188 (21.7%) | 28 (28.3%) | 0.16 |
| Alcohol abuse | 147 (15.2%) | 127 (14.6%) | 20 (20.2%) | 0.18 |
| Cerebral ischemic attacks | 85 (8.8%) | 65 (7.5%) | 20 (20.2%) | <0.001 |
| Transient ischemic attacks | 68 (7.0%) | 53 (6.1%) | 15 (15.2%) | 0.002 |
| Stroke induced hemiplegia | 14 (1.4%) | 11 (1.3%) | 3 (3.0%) | 0.17 |
| Preoperative hypotension | 46 (4.8%) | 25 (2.9%) | 21 (21.2%) | <0.001 |
| Cardiac tamponade | 130 (13.5%) | 75 (8.7%) | 55 (55.6%) | <0.001 |
| Malperfusion syndrome |  |  |  |  |
| Visceral malperfusion | 35 (3.6%) | 27 (3.1%) | 8 (8.1%) | 0.02 |
| Coronary malperfusion | 43 (4.5%) | 33 (3.8%) | 10 (10.1%) | 0.009 |
| Lower limbs malperfusion | 128 (13.3%) | 96 (11.1%) | 32 (32.3%) | <0.001 |
| State of consciousness |  |  |  |  |
| Normal | 951 (98.4%) | 859 (99.1%) | 92 (92.9%) | <0.001 |
| Drowsiness | 10 (1.0%) | 6 (0.7%) | 4 (4.0%) |  |
| Coma | 5 (0.5%) | 2 (0.2%) | 3 (3.0%) |  |
| Intimal dissection entry site |  |  |  |  |
| Ascending aorta | 542 (56.1%) | 487 (56.2%) | 55 (55.6%) | 0.89 |
| Aortic arch | 262 (27.1%) | 234 (27.0%) | 28 (28.3%) |  |
| Descending aorta | 65 (6.7%) | 60 (6.9%) | 5 (5.1%) |  |
| Not found | 97 (10.0%) | 86 (9.9%) | 11 (11.1%) |  |
| Involvement of coronary artery | 201 (20.8%) | 171 (19.7%) | 30 (30.3%) | 0.02 |
| Laboratory tests |  |  |  |  |
| White blood cell count (10^9^/L) | 11.4 (9.1, 13.3) | 11.4 (9.1, 13.2) | 12.2 (9.4, 15.5) | 0.007 |
| Neutrophil count (10^9^/L) | 9.8 (7.6, 11.8) | 9.8 (7.6, 11.5) | 10.7 (7.8, 13.8) | 0.01 |
| Neutrophil ratio (%) | 85.8 (83.0, 89.7) | 85.8 (83.3, 89.8) | 85.8 (82.1, 89.7) | 0.75 |
| Red blood cell count (10^9^/L) | 3.93 (3.49, 4.43) | 3.93 (3.54, 4.44) | 3.83 (3.20, 4.39) | 0.11 |
| Hemoglobin (mg/dL) | 119.6 (105.0, 136.0) | 119.6 (106.0, 136.0) | 116.5 (99.0, 137.0) | 0.17 |
| Platelet count (10^9^/L) | 144.5 (109.8, 172.0) | 147.0 (111.0, 172.0) | 128.0 (86.0, 167.0) | 0.03 |
| Alanine transaminase (U/L) | 25.6 (17.8, 40.4) | 25.6 (17.1, 38.9) | 32.9 (19.8, 105.9) | 0.001 |
| Aspartate transaminase (U/L) | 38.0 (24.0, 62.0) | 37.0 (24.0, 55.4) | 54.2 (33.0, 200.8) | <0.001 |
| Lactate dehydrogenase (U/L) | 452.0 (345.0, 602.0) | 444.2 (331.0, 593.0) | 540.0 (460.0, 968.0) | <0.001 |
| Bilirubin (umol/L) | 16.2 (11.9, 22.1) | 16.2 (11.9, 21.8) | 16.9 (11.9, 25.4) | 0.05 |
| Creatinine (umol/L) | 81.2 (66.0, 105.0) | 81.2 (65.3, 102.0) | 97.2 (69.1, 155.7) | <0.001 |
| Blood urea nitrogen (mmol/L) | 7.3 (6.4, 8.2) | 7.3 (6.3, 8.1) | 7.6 (6.8, 9.1) | 0.01 |
| Creatine kinase-MB (U/L) | 10.5 (7.0, 18.0) | 10.5 (7.0, 18.0) | 17.0 (10.5, 25.0) | <0.001 |
| Cardiac troponin T (ug/L) | 0.025 (0.012, 0.090) | 0.025 (0.011, 0.072) | 0.074 (0.025, 0.386) | <0.001 |
| Brain natriuretic peptide (pg/mL) | 97.0 (44.0, 200.3) | 97.0 (43.3, 198.0) | 97.0 (48.1, 239.0) | 0.56 |
| Prothrombin time (s) | 13.0 (11.8, 14.1) | 12.9 (11.8, 14.0) | 14.0 (12.6, 16.4) | <0.001 |
| Fibrinogen (g/L) | 2.3 (1.7, 2.8) | 2.3 (1.8, 2.8) | 1.8 (1.4, 2.4) | <0.001 |
| D-dimer (mg/L) | 4.96 (3.95, 7.84) | 4.96 (3.91, 7.84) | 5.54 (4.96, 8.42) | 0.02 |

*Abbreviation:* AD=aortic dissection; AVR=aortic valve replacement; CABG=coronary artery bypass graft; CAD=coronary artery disease; COPD=chronic obstructive pulmonary disease; TEVAR=thoracic endovascular aortic repair.

**Table S5. Univariate and multivariate analysis of the risk factors**

| **Variables** | **Univariate analysis** | | | | **Multivariate analysis** | | | | | | | |
| --- | --- | --- | --- | --- | --- | --- | --- | --- | --- | --- | --- | --- |
|  |  |  |  |  | **Model 1** | | | | **Model 2** | | | |
|  | **Odd Ratio** | **95% Confidence Interval** | | **P Value** | **Odd Ratio** | **95% Confidence Interval** | | **P Value** | **Odd Ratio** | **95% Confidence Interval** | | **P Value** |
|  |  | **Lower** | **Upper** |  |  | **Lower** | **Upper** |  |  | **Lower** | **Upper** |  |
| Cerebral ischemic attacks | 3.124 | 1.799 | 5.424 | <0.001 |  |  |  |  |  |  |  |  |
| Transient ischemic attacks | 2.743 | 1.482 | 5.076 | <0.001 |  |  |  |  |  |  |  |  |
| Preoperative hypotension | 9.068 | 4.854 | 16.938 | <0.001 | 7.017 | 3.233 | 15.228 | <0.001 | 6.787 | 3.079 | 14.961 | <0.001 |
| Cardiac tamponade | 13.200 | 8.318 | 20.947 | <0.001 | 15.062 | 8.958 | 25.327 | <0.001 | 14.459 | 8.566 | 24.404 | <0.001 |
| Visceral malperfusion | 2.735 | 1.207 | 6.198 | 0.02 |  |  |  |  |  |  |  |  |
| Coronary malperfusion | 2.840 | 1.354 | 5.955 | 0.009 | 4.832 | 1.963 | 11.890 | 0.001 | 5.146 | 2.072 | 12.783 | <0.001 |
| Lower limbs malperfusion | 3.836 | 2.394 | 6.147 | <0.001 | 4.099 | 2.299 | 7.306 | <0.001 | 4.350 | 2.427 | 7.797 | <0.001 |
| State of consciousness | 4.420 | 2.029 | 9.632 | <0.001 | 3.020 | 1.223 | 7.454 | 0.017 | 3.329 | 1.297 | 8.542 | 0.01 |
| Involvement of coronary artery | 1.770 | 1.117 | 2.804 | 0.02 |  |  |  |  |  |  |  |  |
| White blood cell count | 1.106 | 1.051 | 1.163 | <0.001 |  |  |  |  |  |  |  |  |
| Neutrophil count | 1.099 | 1.040 | 1.161 | 0.001 |  |  |  |  |  |  |  |  |
| Bilirubin | 1.015 | 1.004 | 1.027 | 0.009 |  |  |  |  | 1.015 | 1.001 | 1.029 | 0.03 |
| Blood urea nitrogen | 1.050 | 1.005 | 1.097 | 0.03 |  |  |  |  | 1.061 | 1.002 | 1.124 | 0.04 |
| Cardiac troponin T | 1.268 | 1.101 | 1.459 | 0.001 |  |  |  |  |  |  |  |  |
| Prothrombin time | 1.101 | 1.050 | 1.153 | <0.001 |  |  |  |  |  |  |  |  |
| Fibrinogen | 0.688 | 0.545 | 0.869 | 0.002 |  |  |  |  |  |  |  |  |

**Table S6. Intraoperative and postoperative variables of low- and high-risk aTAAD patients**

|  | **Low-risk patients** | | | | **High-risk patients** | | | | **P3 Value** |
| --- | --- | --- | --- | --- | --- | --- | --- | --- | --- |
|  | **All patients**  **N=920** | **Survival**  **N=827** | **Death**  **N=93** | **P1 Value** | **All patients**  **N=444** | **Survival**  **N=347** | **Death**  **N=97** | **P2 Value** |  |
| Intraoperative variables |  |  |  |  |  |  |  |  |  |
| Operation time (hour) | 7.8±1.9 | 7.7±1.7 | 9.3±2.4 | <0.001 | 7.9±2.2 | 7.6±2.0 | 9.0±2.6 | <0.001 | 0.412 |
| CPB time (min) | 231.7±72.3 | 228.0±67.8 | 264.6±99.8 | 0.001 | 240.5±80.6 | 230.0±66.9 | 278.5±109.7 | 0.001 | 0.051 |
| ACC time (min) | 161.4±52.1 | 159.3±51.2 | 180.2±56.6 | <0.001 | 168.6±59.6 | 164.4±53.8 | 183.9±75.4 | 0.045 | 0.030 |
| Arterial cannulation approach | | | | | | | | | |
| Ascending aorta | 15 (1.6%) | 13 (86.7%) | 2 (13.3%) | 0.610 | 5 (1.1%) | 3 (60.0%) | 2 (40.0%) | 0.048 | 0.461 |
| Femoral artery | 211 (22.9%) | 193 (91.5%) | 18 (8.5%) |  | 118 (26.6%) | 86 (72.9%) | 32 (27.1%) |  |  |
| Axillary artery | 190 (20.7%) | 173 (91.1%) | 17 (8.9%) |  | 86 (19.4%) | 75 (87.2%) | 11 (12.8%) |  |  |
| Axillary-Femoral artery | 504 (54.8%) | 448 (88.9%) | 56 (11.1%) |  | 235 (52.9%) | 183 (77.9%) | 52 (22.1%) |  |  |
| DHCA | 907 (98.6%) | 815 (98.5%) | 92 (98.9%) | 1.000 | 438 (98.6%) | 341 (98.3%) | 97 (100.0%) | 0.347 | 1.000 |
| Cerebral perfusion | 792 (86.1%) | 708 (85.6%) | 84 (90.3%) | 0.268 | 354 (79.7%) | 283 (81.6%) | 71 (73.2%) | 0.086 | 0.003 |
| None | 128 (13.9%) | 119 (93.0%) | 9 (7.0%) | 0.423 | 90 (20.3%) | 64 (71.1%) | 26 (28.9%) | 0.166 | 0.010 |
| Anterograde | 744 (80.9%) | 666 (89.5%) | 78 (10.5%) |  | 334 (75.2%) | 268 (80.2%) | 66 (19.8%） |  |  |
| Retrograde | 48 (5.2%) | 42 (87.5%) | 6 (12.5%) |  | 20 (4.5%) | 15 (75.0%) | 5 (25.0%) |  |  |
| Cerebral perfusion time (min) | 29.7±10.9 | 29.5±11.0 | 32.1±10.0 | 0.043 | 30.2±11.3 | 29.9±11.1 | 31.4±12.0 | 0.281 | 0.433 |
| Lowest hypothermia temperature (℃) | 21.0 (20.0, 22.0) | 21.0 (20.0, 22.0) | 20.0 (18.0, 22.0) | 0.058 | 21.5 (20.0, 23.0) | 22.0 (20.0, 24.0) | 20.0 (18.0, 22.0) | 0.007 | 0.078 |
| Root treatment |  |  |  |  |  |  |  |  |  |
| Untreated | 9 (1.0%) | 8 (88.9%) | 1 (11.1%) | 0.693 | 5 (1.1%) | 4 (80.0%) | 1 (20.0%) | 0.377 | 0.918 |
| Root repair | 699 (76.0%) | 631 (90.3%) | 68 (9.7%) |  | 335 (75.5%) | 257 (76.7%) | 78 (23.3%) |  |  |
| Root replacement | 212 (23.0%) | 189 (89.2%) | 23 (10.8%) |  | 104 (23.4%) | 86 (82.7%) | 18 (17.3%) |  |  |
| Arch treatment |  |  |  |  |  |  |  |  |  |
| Untreated | 0 (0.0%) | 0 (0.0%) | 0 (0.0%) | 0.757 | 5 (1.1%) | 5 (1.4%) | 0 (0.0%) | <0.001 | <0.001 |
| Hemi-arch | 166 (18.0%) | 151 (91.0%) | 15 (9.0%) |  | 107 (24.1%) | 83 (77.6%) | 24 (22.4%) |  |  |
| MiTAR | 108 (11.7%) | 96 (88.9%) | 12 (11.1%) |  | 52 (11.7%) | 43 (82.7%) | 9 (17.3%) |  |  |
| TAR | 335 (36.4%) | 296 (88.4%) | 39 (11.6%) |  | 127 (28.6%) | 97 (76.4%) | 30 (23.6%) |  |  |
| Branch stent | 32 (3.5%) | 29 (90.6%) | 3 (9.4%) |  | 8 (1.8%) | 6 (75.0%) | 2 (25.0%) |  |  |
| Fenestrated stent | 279 (30.3%) | 255 (91.4%) | 24 (8.6%) |  | 145 (32.7%) | 113 (77.9%) | 32 (22.1%) |  |  |
| Limited arch replacement | 585 (63.6%) | 531 (90.8%) | 54 (9.2%) | 0.257 | 317 (71.4%) | 250 (78.9%) | 67 (21.1%) | 0.612 | 0.005 |
| Extended arch replacement | 335 (36.4%) | 296 (88.4%) | 39 (11.6%) |  | 127 (28.6%) | 97 (76.4%) | 30 (23.6%) |  |  |
| Stents |  |  |  |  |  |  |  |  |  |
| Without stent | 154 (16.7%) | 140 (90.0%) | 14 (9.1%) | 0.809 | 105 (23.6%) | 81 (77.1%) | 24 (22.9%) | 0.945 | 0.009 |
| FET | 407 (44.2%) | 363 (89.2%) | 44 (10.8%) |  | 185 (41.7%) | 146 (78.9%) | 39 (21.1%) |  |  |
| Stent in arch | 359 (39.0%) | 324 (90.3%) | 35 (9.7%) |  | 154 (34.7%) | 120 (77.9%) | 34 (22.1%) |  |  |
| CABG | 54 (5.9%) | 39 (4.7%) | 15 (16.1%) | <0.001 | 39 (8.9%) | 21 (6.1%) | 18 (18.6%) | <0.001 | 0.051 |
| Planned CABG | 37 (68.5%) | 32 (86.5%) | 5 (13.5%) | 0.001 | 28 (71.8%) | 18 (64.3%) | 10 (35.7%) | 0.072 | 0.821 |
| Unplanned CABG | 17 (31.5%) | 7 (41.2%) | 10 (58.8%) |  | 11 (28.2%) | 3 (27.3%) | 8 (72.7%) |  |  |
| Mitral valve replacement | 6 (0.7%) | 4 (0.5%) | 2 (2.2%) | 0.116 | 1 (0.2%) | 1 (0.3%) | 0 (0.0%) | 1.000 | 0.438 |
| Mitral valve repair | 15 (1.6%) | 12 (1.5%) | 3 (3.2%) | 0.187 | 3 (0.7%) | 2 (0.6%) | 1 (1.0%) | 0.524 | 0.206 |
| Tricuspid valve repair | 18 (2.0%) | 15 (1.8%) | 3 (3.2%) | 0.415 | 4 (0.9%) | 4 (1.2%) | 0 (0.0%) | 0.581 | 0.174 |
| AFRA | 5 (0.5%) | 4 (0.5%) | 1 (1.1%) | 0.414 | 0 (0.0%) | 0 (0.0%) | 0 (0.0%) | - | 0.180 |
| Postoperative variables |  |  |  |  |  |  |  |  |  |
| Cerebral complications | 62 (6.7%) | 51 (6.2%) | 11 (11.8%) | 0.048 | 43 (9.7%) | 27 (7.8%) | 16 (16.5%) | 0.013 | 0.065 |
| Postoperative stroke | 46 (5.0%) | 38 (4.6%) | 8 (8.8%) | 0.125 | 30 (6.8%) | 19 (5.5%) | 11 (11.3%) | 0.064 | 0.208 |
| Postoperative hemiplegia | 17 (1.8%) | 16 (1.9%) | 1 (1.1%) | 1.000 | 11 (2.5%) | 7 (2.0%) | 4 (4.1%) | 0.266 | 0.541 |
| Mechanical ventilation time (hour) | 23.6 (13.3, 70.0) | 22.0 (13.0, 70.0) | 41.5 (20.0, 120.0) | 0.001 | 23.6 (13.3, 70.0) | 31.0 (13.0, 68.0) | 66.0 (24.0, 120.0) | 0.001 | 0.738 |
| Re-intubation | 59 (6.4%) | 45 (5.4%) | 14 (15.1%) | 0.001 | 27 (6.1%) | 16 (4.6%) | 11 (11.3%) | 0.019 | 0.905 |
| Tracheotomy | 36 (3.9%) | 30 (3.6%) | 6 (6.5%) | 0.251 | 20 (4.5%) | 17 (4.9%) | 3 (3.1%) | 0.586 | 0.662 |
| CRRT establishment | 95 (10.3%) | 71 (8.6%) | 24 (25.8%) | <0.001 | 72 (16.2%) | 51 (14.7%) | 21 (21.6%) | 0.119 | 0.002 |
| Secondary thoracotomy | 52 (5.7%) | 44 (5.3%) | 8 (8.6%) | 0.231 | 24 (5.4%) | 16 (4.6%) | 8 (8.2%) | 0.201 | 0.900 |
| Surgical infection | 24 (2.6%) | 17 (2.1%) | 7 (7.5%) | 0.007 | 12 (2.7%) | 6 (1.7%) | 6 (6.2%) | 0.028 | 1.000 |
| Postoperative lower limbs malperfusion | 9 (1.0%) | 9 (1.1%) | 0 (0.0%) | 0.610 | 16 (3.6%) | 8 (2.3%) | 8 (8.2%) | 0.011 | 0.001 |
| Postoperative visceral malperfusion | 9 (1.0%) | 7 (0.8%) | 2 (2.2%) | 0.229 | 14 (3.2%) | 6 (1.7%) | 8 (8.2%) | 0.004 | 0.005 |
| Postoperative gastrointestinal bleeding | 7 (0.8%) | 7 (0.8%) | 0 (0.0%) | 1.000 | 7 (1.6%) | 4 (1.2%) | 3 (3.1%) | 0.180 | 0.164 |

*Abbreviation:* AAC=aortic cross-clamping; AFRA=atrial fibrillation radiofrequency ablation; CABG=coronary artery bypass graft; CPB=cardiopulmonary bypass; CRRT=continuous renal replacement therapy; DHCA=deep hypothermic circulatory arrest; FET=frozen elephant trunk; MiTAR=modified “*in situ*” total arch replacement; TAR=total arch replacement. (P1 and P2 mean the statistic difference between survival and dead patients in the low- and high-risk group separately. P3 means the statistic difference of all patients between low- and high-risk group.)

**Table S7. Univariate analysis of postoperative mortality in low- and high-risk patients**

| **Variables** | **Low-risk patients** | | | | **High-risk patients** | | | |
| --- | --- | --- | --- | --- | --- | --- | --- | --- |
|  | **Odd ratio** | **95% Confidence Interval** | | **P Value** | **Odd ratio** | **95% Confidence Interval** | | **P Value** |
|  |  | **Lower** | **Upper** |  |  | **Lower** | **Upper** |  |
| **Intraoperative variables** |  |  |  |  |  |  |  |  |
| Operation time | 1.512 | 1.353 | 1.689 | <0.001 | 1.322 | 1.192 | 1.467 | <0.001 |
| CPB time | 1.005 | 1.003 | 1.008 | <0.001 | 1.007 | 1.004 | 1.010 | <0.001 |
| ACC time | 1.007 | 1.003 | 1.011 | <0.001 | 1.005 | 1.002 | 1.009 | 0.005 |
| Axillary artery cannulation | - | - | - | - | 0.464 | 0.236 | 0.914 | 0.029 |
| Lowest hypothermia temperature | - | - | - | - | 1.122 | 1.021 | 1.233 | 0.016 |
| CABG | 3.116 | 2.026 | 4.792 | <0.001 | 3.537 | 1.799 | 6.953 | <0.001 |
| **Postoperative variables** |  |  |  |  |  |  |  |  |
| Cerebral complications | 2.041 | 1.024 | 4.070 | 0.048 | 2.341 | 1.204 | 4.551 | 0.013 |
| Mechanical ventilation time | - | - | - | - | 1.005 | 1.002 | 1.008 | 0.003 |
| Re-intubation | 3.080 | 1.619 | 5.857 | 0.001 | 2.646 | 1.185 | 5.910 | 0.019 |
| CRRT establishment | 3.704 | 2.192 | 6.257 | <0.001 |  |  |  |  |
| Surgical infection | 3.878 | 1.564 | 9.614 | 0.007 | 3.747 | 1.181 | 11.894 | 0.028 |
| Postoperative lower limbs malperfusion | - | - | - | - | 3.809 | 1.391 | 10.431 | 0.011 |
| Postoperative visceral malperfusion | - | - | - | - | 5.109 | 1.728 | 15.102 | 0.004 |

*Abbreviation:* AAC=aortic cross-clamping; CABG=coronary artery bypass graft; CPB=cardiopulmonary bypass; CRRT= continuous renal replacement therapy.

**Table S8. Postoperative complications of different artery cannulation approach in high-risk patients**

| **Variables** | **Axillary artery**  **N=86** | **Femoral artery**  **N=118** | **Axillary-Femoral artery**  **N=235** | **P1 value** | **P2 value** |
| --- | --- | --- | --- | --- | --- |
| Cerebral complications | 6 (7.0%) | 12 (10.2%) | 24 (10.2%) | 0.466 | 0.402 |
| Postoperative stroke | 3 (3.5%) | 7 (5.9%) | 19 (8.1%) | 0.524 | 0.212 |
| Postoperative hemiplegia | 2 (2.3%) | 5 (4.2%) | 4 (1.7%) | 0.701 | 0.661 |
| CRRT establishment | 8 (9.3%) | 20 (16.9%) | 43 (18.3%) | 0.150 | 0.058 |
| Postoperative lower limbs malperfusion | 1 (1.2%) | 5 (4.2%) | 10 (4.3%) | 0.404 | 0.300 |
| Postoperative visceral malperfusion | 1 (1.2%) | 2 (1.7%) | 11 (4.7%) | 1.000 | 0.193 |
| Postoperative gastrointestinal bleeding | 2 (2.3%) | 3 (2.5%) | 2 (0.9%) | 1.000 | 0.292 |

*Abbreviation:* CRRT= continuous renal replacement therapy. P1 means the statistic difference between axillary artery and femoral artery. P2 means the statistic difference between axillary artery and axillary-femoral artery.
